# Men, women…who cares? A population-based study on sex differences and gender roles in empathy and moral cognition

**DOI:** 10.1371/journal.pone.0179336

**Published:** 2017-06-20

**Authors:** Sandra Baez, Daniel Flichtentrei, María Prats, Ricardo Mastandueno, Adolfo M. García, Marcelo Cetkovich, Agustín Ibáñez

**Affiliations:** 1 Laboratory of Experimental Psychology and Neuroscience (LPEN), Institute of Cognitive and Translational Neuroscience (INCyT), INECO Foundation, Favaloro University, Buenos Aires, Argentina; 2 Departamento de Psicología, Universidad de los Andes, Bogotá, Colombia; 3 Grupo de Investigación Cerebro y Cognición Social, Bogotá, Colombia; 4 National Scientific and Technical Research Council (CONICET), Buenos Aires, Argentina; 5 Intra*Med*, Buenos Aires, Argentina; 6 Faculty of Elementary and Special Education (FEEyE), National University of Cuyo (UNCuyo), Mendoza, Argentina; 7 Universidad Autónoma del Caribe, Barranquilla, Colombia; 8 Center for Social and Cognitive Neuroscience (CSCN), School of Psychology, Adolfo Ibáñez University, Santiago de Chile, Chile; 9 Australian Research Council Centre of Excellence in Cognition and its Disorders, Sydney, Australia; Universitatsklinikum Tubingen, GERMANY

## Abstract

Research on sex differences in empathy has revealed mixed findings. Whereas experimental and neuropsychological measures show no consistent sex effect, self-report data consistently indicates greater empathy in women. However, available results mainly come from separate populations with relatively small samples, which may inflate effect sizes and hinder comparability between both empirical corpora. To elucidate the issue, we conducted two large-scale studies. First, we examined whether sex differences emerge in a large population-based sample (*n* = 10,802) when empathy is measured with an experimental empathy-for-pain paradigm. Moreover, we investigated the relationship between empathy and moral judgment. In the second study, a subsample (*n* = 334) completed a self-report empathy questionnaire. Results showed some sex differences in the experimental paradigm, but with minuscule effect sizes. Conversely, women did portray themselves as more empathic through self-reports. In addition, utilitarian responses to moral dilemmas were less frequent in women, although these differences also had small effect sizes. These findings suggest that sex differences in empathy are highly driven by the assessment measure. In particular, self-reports may induce biases leading individuals to assume gender-role stereotypes. Awareness of the role of measurement instruments in this field may hone our understanding of the links between empathy, sex differences, and gender roles.

## Introduction

“Boys will be boys”, “girls are emotional and sensitive”, “men don’t cry, women do”. These and other gender stereotypes have perpetuated the notion that women are more empathetic and caring than men. Throughout, the term “gender” is used to refer to “the attitudes, feelings, and behaviors that a given culture associates with a person’s biological sex”[1], while the term “sex” refers to a person’s biological status [1]. Supporting evidence for gender stereotypes has been obtained through self-report empathy questionnaires [2–5], which may be strongly biased by gender-relevant social expectations [6, 7]. Similarly, results from self-administered measures have motivated the view that women are more care-oriented than men in moral reasoning [8, 9]. While this evidence seems to reveal sex differences in both empathy and moral judgment, it stems from instruments likely to bias responses towards gender-role stereotypes [7, 10, 11]. Indeed, sex differences are typically absent in relevant experimental tasks [4, 12] and physiological measures [6, 13, 14]. Moreover, available results stem from relatively small samples, which casts doubts on both lines of research because they may inflate effect sizes and yield inaccurate estimations of the relevance of a significant difference. Through the present population-based study, we aimed to (a) assess whether empathy, moral judgment, and their relationship differ between sexes; and (b) test whether previously reported empathy differences obtained though gender-role-biased instruments also emerge on a well-validated experimental paradigm [15–19]. In particular, by analyzing data from massive samples, we aim to go beyond *p*-values as indicators of significance and focus on the systematicity of potential differences by considering truly informative effect sizes.

Previous studies on sex differences in empathy have yielded mixed results. Such differences are stronger when empathy is measured with self-report questionnaires (e.g., [2, 3–5, 20–22]). Sex differences favoring women were also observed through a task assessing feelings of sympathy towards targets, prior to performing an empathic accuracy task [23]. Contrarily, no sex differences emerge when empathy is assessed with experimental tasks [4, 12] or physiological measures [6, 13, 14]. Thus, sex differences in empathy vary dramatically depending on the method of assessment. Such inconsistencies have been further fueled by other factors. For instance, available results stem from relatively small samples and no population-based studies have been performed. In addition, although reporting confidence intervals is a highly desirable practice in psychology [24], it has been overlooked in most previous studies. Most of the extant work on sex differences in empathy has been based on formal statistical significance–typically, a *p*-value less than .05. *P-values* alone do not permit any direct statement about the direction or size of a difference between groups. For this purpose, confidence intervals provide information about statistical significance, as well as the direction and strength of the effect [25]. Moreover confidence intervals help to combine evidence over experiments and give information about precision [24]. A further caveat is that although effect sizes are considered the most important outcome of empirical studies [26], they are absent in most available research (e.g., [2, 3, 4, 22, 27]). In this sense, the statistical power of psychological and neuroscientific studies is typically undermined by the use of small samples, which leads to overestimated effect sizes [26, 28–33]. Statistical power depends on the sample size of the study, the effect size, and the significance criterion [26]. As the sample size increases, so does the reliability of the sample values and the extent to which such values can be expected to provide accurate estimations of the population’s values [26]. Consequently, larger sample sizes increase power and decrease estimation error [34]. In addition, data from population-based sample sizes increase the precision of estimated effects [29, 35–37], although they also increase the likelihood of obtaining unduly significant *p*-values. Accordingly, large-scale population-based studies reporting confidence intervals and effect sizes stand out by the robustness of their results and the precision of their estimates: even if their significance levels cannot be fully informative, the size of the reported effects can offer precise estimations of the relevance of apparent differences.”

Self-report measures of empathy may be strongly influenced by gender-relevant social norms and expectations [6, 7, 21, 23]. Indeed, self-reported empathy has been linked with social desirability [38–40], and sex differences in such a measure correlate better with gender roles than with biological sex [41]. As emotionality and sensitivity are both part of the stereotypical feminine role, women could be more willing than males to portray themselves as empathic, even if empathic responsiveness were similar for both groups [7]. Supporting this notion, reports on stereotypes [42, 43] show that both women and men endorse the generalizations that the former are more “sensitive to the feelings of others” and have more “emotional insight than men”. These traditional gender stereotypes still persist, as women, relative to men, continue to be regarded as warmer, nicer, and more sensitive, modest, and sociable than men [44, 45]. Together with behavioral [4, 12] and physiological [6, 13, 14] empathy studies yielding no sex-related effects, the evidence suggests that sex differences in this domain may emerge only when gender-role stereotypes are activated by explicit self-assessment instruments [7, 10, 46].

A similar scenario concerns moral judgment. For instance, men and women have been argued to differentially favor justice-oriented and care-oriented moral reasoning, respectively (Gilligan, 1982; Gilligan & Attanucci, 1988). Also, moral dilemmas have been reported to yield more utilitarian responses in men than in women [47, 48]. However, some of these results might also be biased by the use of self-report instruments [11].

The relationship between empathy and morality has been well established [49–53]. For instance, empathy-related processes are thought to motivate prosocial behavior and caring for others. Also, empathy could both provide a foundation for morality [49, 50, 52] and interfere with morally adequate practices–for instance, by introducing partiality towards in-group members [49]. Additional support for a link between both domains is provided by previous studies [54, 55] showing that low empathic concern levels predict utilitarian moral judgment.

Considering these caveats, and in light of the tight links between empathy and morality, we conducted two large population-based studies to assess whether sex differences in each domain and in their relations prove instrument-dependent. In Study 1, we employed an experimental empathy-for-pain task (EPT) alongside two well-known moral judgment tasks. Empathy-for-pain paradigms reliably induce empathic responses [50, 52, 56, 57], engaging putative neural circuits [58] and triggering automatic sensorimotor resonance between other and self [59–61]. Importantly, relative to self-report instruments, these tasks can induce more automatic responses [60, 61] which are less likely to be influenced by gender-relevant social stereotypes and expectations. Questions included in the EPT are aimed at assessing the emotional responses to others in pain as well as aspects related to the action’s moral evaluation. This task does not include explicit questions regarding the individuals’ empathic abilities or attitudes towards empathy. While in self-report questionnaires individuals are asked to rate themselves on empathic behaviors and affective responses, in the EPT they are asked to rate their emotional responses to others’ behaviors in different hypothetical scenarios [16–18, 62]. Questions included in the EPT are less explicitly associated with empathy and are therefore less likely to elicit a social desirability bias. Thus, this instrument does not involve a self-assessment on empathic abilities, nor does it activate the gender-role stereotype that women are more empathetic than men.

In Study 2, we aimed to (a) replicate previously reported sex differences in self-reported empathy and (b) test whether results obtained in Study 1 were replicated when subjective data are considered. To these ends, a subsample of the subjects from Study 1 completed a questionnaire on various aspects of empathy. Given that responses to self-report instruments are typically biased to social desirability and gender-role stereotypes, we expected sex differences to become manifest only in this second study.

## Materials and methods

### Participants

Study 1 comprised 10,802 individuals (5,365 women and 5,437 men) with a mean age of 37.64 (*SD* = 12.51). Study 2 was based on a subsample of 334 subjects (164 women, 170 men) with a mean age of 46.55 (*SD* = 10.56). All participants were university students and professionals possessing at least 12 years of education (see details on participants nationality in S1 Fig). Participants were recruited via the online portal Intramed (www.intramed.net). This recruitment procedure has been employed in several studies (e.g., [63, 64, 65]). A banner was placed in each user’s home page, inviting visitors to voluntarily access an online survey. Participants were excluded from analysis if they did not complete the survey. In addition, participants who completed the entire survey in an extremely short time (less than 3 minutes) were excluded as this suggests that they did not paid full attention to the tasks. According with the reaction times measured in our previous studies [16–18, 62], it would be impossible for a subject to read, understand and complete both the EPT and the moral judgment tasks in less than 3 minutes. All participants gave their informed consent in accordance with the Declaration of Helsinki by pressing an “I agree” button located beneath an explanatory letter. Potential respondents were informed of the anonymity of their responses. The Ethics Committee of the Institute of Cognitive Neurology approved this study.

### Procedure and instruments

#### Study 1

Participants first reported their age, gender, occupation, and country of residence and then completed a series of tasks, as described below.

Empathy for pain was assessed with a modified version of a task previously employed in several studies of our group [15–19, 62], which evaluates various aspects of empathy in events involving either intentional or accidental harm. Stimuli consisted in 11 animated scenarios (4 intentional, 4 accidental, 3 neutral) featuring two individuals. Motion was implied in each scenario by the successive presentation of three digital color pictures. The durations of the first, second, and third pictures in each animation were 500, 200, and 1000 ms, respectively. In the intentional harm scenarios, one person deliberately inflicted pain on another (e.g., by purposely stepping on his/her toe). In the accidental harm scenarios, one person accidentally inflicted pain on another (e.g., by hitting him/her with a bat). In the neutral scenarios, both persons interacted in the absence of pain (e.g., by exchanging flowers).

Importantly, since the protagonists’ faces were not visible, facial emotional reactions were factored out from the task. However, body expressions and postures provided sufficient information about the victim’s emotional reaction and the agent’s intention. In this abbreviated version, participants respond to five questions evaluating: (a) comprehension of the agent’s intention (was the action done on purpose?), (b) empathic concern (how sad do you feel for the victim?), (c) degree of discomfort (how upset do you feel for what happened in the situation?), (d) intention to harm (how bad was the agent’s intention?), and (e) punishment (how much penalty does this action deserve?). Participants respond to these same five questions after viewing each scenario. The question about purpose was answered by selecting “Yes” or “No”. The other questions were answered using a visual analogue scale ranging from 0 to 100 –these numbers were not visible to participants. The meaning of the scale extremes depended on the question. For example, in the question “how sad do you feel for the victim?”, one extreme of the bar reads “I feel very sad” and the other extreme reads “I don’t feel sad at all”. We measured accuracy for the agent’s intention question and ratings for the other questions. Before testing, participants familiarized with the task by completing a training trial.

This task is based on the comparison of three different types of scenarios (accidental harm, intentional harm, and neutral situations). This and similar versions of the EPT have been employed in numerous behavioral [16, 18, 62, 66] and neuroimaging [17, 50] studies in different countries assessing clinical (e.g., [15, 16–19, 62]) and non-clinical populations (e.g., [50, 52, 58]). The ensuing results systematically show that responses to each question are modulated by the context in which the action occurs. Specifically, empathy ratings (i.e., empathic concern, discomfort, intention to hurt, and punishment) are higher for intentional harm than for accidental harm, and ratings for these kinds of scenarios are higher than those for neutral situations. If the format of the question may induce any response bias, this would equally affect the three conditions. Thus, it is unlikely that differences in empathy ratings among stimulus types are explained by biased responses.

In addition, participants were presented with two moral dilemmas [67, 68] in which they had to choose whether they would harm one person to save another five. On the one hand, in the standard trolley dilemma (impersonal), participants had to decide whether they would flip a switch to redirect a trolley onto a man in order to save five other individuals. Such a choice is considered a utilitarian response, whereas a refrain from flipping the switch is deemed non-utilitarian. On the other hand, in footbridge dilemma (personal) participants also had the chance to save five people, but this time by pushing a man off a bridge in order to stop a trolley from hitting them further down the tracks. Accepting to push the man constitutes a utilitarian response, whereas failure to do so is regarded as a non-utilitarian decision. In addition, for comparison purposes, we included a non-moral dilemma in which participants had to choose whether to travel by bus or train given certain time constraints.

Importantly, the level of personal engagement and the form of inflicted harm is different in each moral dilemma: whereas the trolley dilemma proves more impersonal and less emotionally salient, the footbridge dilemma involves more personal engagement and greater emotionally salience [69, 70]. Also, although both moral dilemmas are logically equivalent, the impersonal one does not require consideration of an emotion-evoking personal violation to reach a utilitarian outcome [69, 70]. Thus, the vast majority of individuals select the utilitarian option in the trolley dilemma and the non-utilitarian option in the footbridge dilemma [71–73]. Finally, more than 80% of participants typically provide positive responses in the non-moral dilemma [72].

#### Study 2

In addition to the measures described above, the subsample participating in Study 2 completed a self-report questionnaire of empathy, as detailed below.

Participants completed the Interpersonal Reactivity Index (IRI), a 28-item self-report questionnaire that separately measures cognitive and affective components of empathy [27]. The instrument contains four scales, each tapping a specific subdomain, namely: (a) empathic concern (the tendency to experience feelings of warmth, compassion, and concern for other people), (b) personal distress (one’s own feelings of personal unease and discomfort in reaction to the emotions of others), (c) perspective taking (the tendency to adopt the point of view of other people), and (d) fantasy (the tendency to imagine oneself as experiencing the feelings and actions of fictitious characters). Each subscale has seven items measured on a 5-point Likert scale ranging from 0 (“does not describe me well”) to 4 (“describes me very well”). A total score can also calculated by adding the scores of the four subscales.

### Data analysis

#### Study 1

Demographic and neuropsychological data were compared between samples with ANOVA tests; categorical variables were analyzed through X^2^ tests. The assumption of normality was verified using the Shapiro-Wilk test. Following previous procedures [15–18, 62], we separately analyzed the ratings for each question of the EPT using a 2 (group: female vs. male) by 3 (stimulus type: intentional, accidental, neutral) factorial ANOVA. Tukey’s HSD post-hoc tests were used to further scrutinize differences among stimulus type (intentional harm, accidental harm, and neutral situations) for each rating (purpose comprehension, empathic concern, discomfort, intention to hurt, and punishment) and significant interactions between group and stimulus type. Between-group differences in responses to the moral dilemmas and the non-moral dilemma were explored using X^2^ tests.

We re-analyzed the empathy data using the responses to the personal dilemma as a covariate–for a similar approach see [16, 74]. Finally, we conducted multiple regression analyses to explore whether EPT performance was explained by sex or moral judgments.

Considering our large sample sizes, between-group differences in both studies were reported with *p*-values and their associated effect sizes. The statistical significance level was set at *p* < .05. Effect sizes were calculated through partial eta (η^2^) tests. Following Cohen’s classification of effect sizes [75], we considered effects to be statistically relevant at η^2^ ≥ 0.01 (i.e., small or higher effect sizes). Effect sizes for X^2^ tests were calculated through Cramer’s V. Values ≥ 0.1 (i.e., small or higher effect sizes) were considered statistically relevant [75]. Moreover, to mitigate the inflated *p*-value problem (characteristic of large sample sizes) and test the consistency of sex differences across different sample sizes, all statistical analyses were also performed considering the subsample reported in Study 2 (*n* = 334). Details of these results can be found in S2 Text.

#### Study 2

Demographic data was analyzed following the same procedures as described for Study 1. The assumption of normality was verified using the Shapiro-Wilk test. Sex differences in self-reported empathy were tested through one-way ANOVA tests. As in Study 1, we re-analyzed self-reported empathy data using the response to the personal dilemma as covariate. Finally, as in Study 1, we conducted multiple regression analyses to explore whether IRI scores were explained by sex or moral judgment. For these analyses, the items yielding significant sex differences were framed as dependent variables.

## Results of Study 1

Study 1 comprised 10,802 individuals who first reported their age, gender, occupation, and country of residence and then completed the EPT and a moral judgment task. No significant age differences were observed between women and men (*F*(1, 10800) = 0.20, *p =* .65, η^2^ = 0.00001).

### Empathy for pain

**Fig 1:**
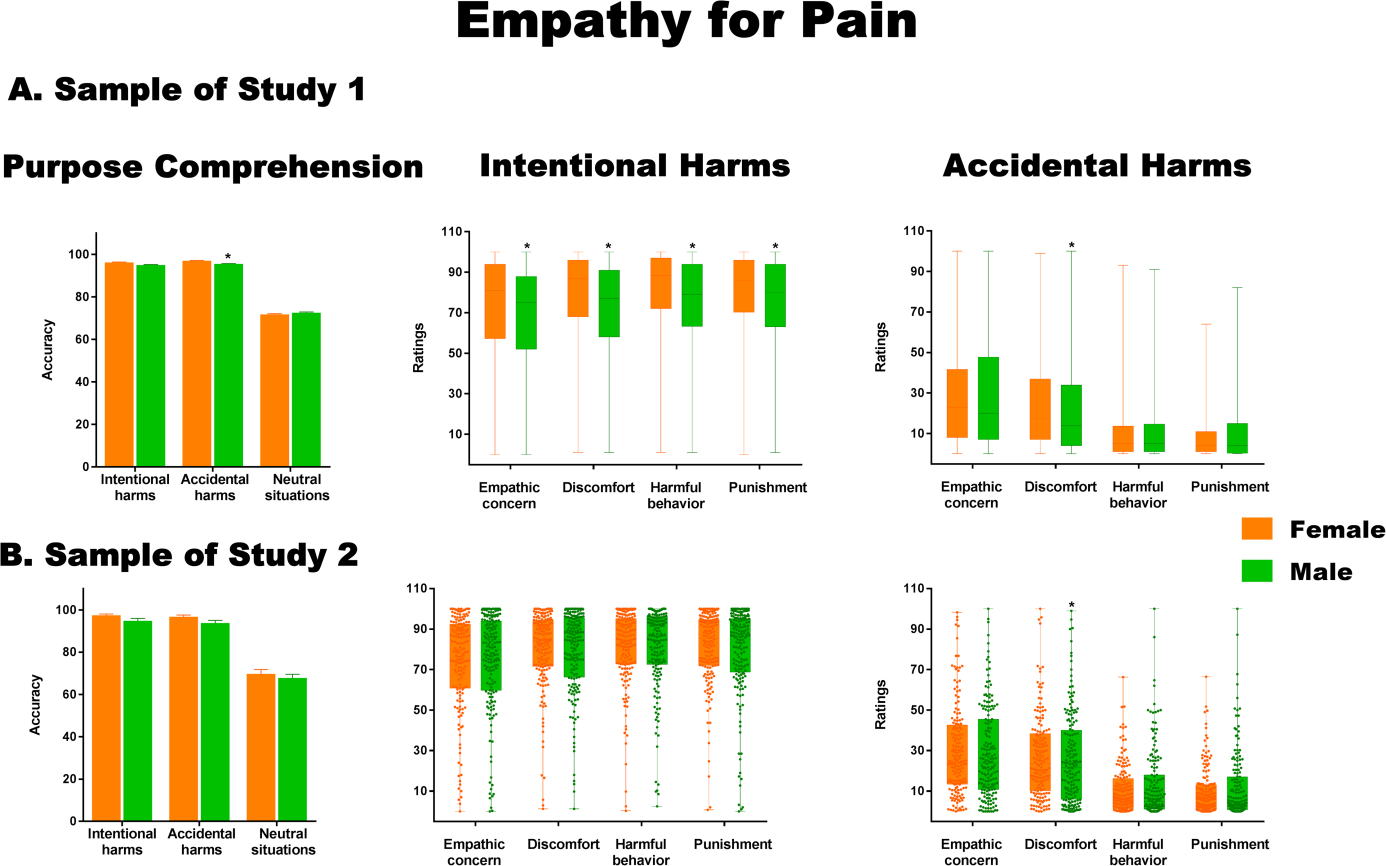
Significant sex differences in empathy-for-pain ratings. (A) Results from the sample of Study 1. (B) Results from the sample of Study 2. Asterisks indicate significant differences (*p* < .05).

The five EPT measures (see methods section) revealed significant between-group differences. These are shown in Fig 1A and detailed below. Descriptive statistical data are provided in S1 Table.

A main effect of sex emerged in intention comprehension (*F*(1, 10800 = 4.56, *p <* .05, η^2^ = 0.0004), with greater accuracy for women than men. We also observed a significant interaction between sex and stimulus type (*F*(2, 21600 = 10.57, *p <* .001, η^2^ = 0.0009). A post-hoc analysis (Tukey’s HSD, *MS* = 281.51, *df* = 32224) revealed that women were more accurate than men in ascertaining the agent’s intention situations of accidental harm (*p* < .05).

A main effect of sex was also observed in empathic concern (*F*(1, 10800 = 11.88, *p <* .001, η^2^ = 0.001), showing higher ratings in women. Moreover, a significant interaction between sex and stimulus type emerged (*F*(2, 21600) = 17.50, *p <* .001, η^2^ = 0.001). A post-hoc analysis (Tukey HSD, *MS* = 463.06, *df* = 26139) showed that women provided higher empathic concern ratings than men for intentional harm (*p <* .001).

In addition, women showed higher discomfort ratings than men [main effect of sex: (*F*(1, 10800 = 35.93, *p <* .001, η^2^ = 0.003)]. For these ratings, we also found a significant interaction between sex and stimulus type (*F*(2, 21600) = 29.50, *p <* .002, η^2^ = 0.003). A post-hoc analysis (Tukey HSD, *MS* = 374.26, *df* = 28121) revealed higher discomfort for intentional (*p <* .001) and accidental (*p <* .001) harm for women than men.

With respect to intention to harm, there was a main affect of sex(*F*(1, 10800 = 3.56, *p <* .05, η^2^ = 0.0003), showing higher ratings in women. There was also a significant interaction between sex and stimulus type (*F*(2, 21600) = 32.81, *p <* .001, η^2^ = 0.003). A post-hoc analysis (Tukey’s HSD, *MS* = 252.28, *df* = 31182) showed higher intention-to-harm ratings for intentional harms in women relative to men (*p <* .001).

Furthermore, women gave higher punishment ratings than men [main effect of sex: (*F*(1, 10800 = 4.47, *p <* .05, η^2^ = 0.0004)]. A significant interaction between sex and stimulus type (*F*(2, 21600 = 33.33, *p <* .001, η^2^ = 0.003) was also observed. A post-hoc analysis (Tukey’s HSD, *MS* = 256.80, *df* = 31075) revealed higher punishment ratings for intentional harms in women than in men (*p <* .001).

All data in which we found significant differences between women and men, were later reanalyzed with Kruskal-Wallis tests. All results remained unchanged (see S1 Text).

In order to rule out trivial significant differences related to the large sample size, we re-analyzed the EPT data in the subsample of Study 2. A radically different picture emerged. There was a main effect of sex (*F*(1, 332 = 6.21, *p <* 0.01, η^2^ = .001) in purpose comprehension, with higher accuracy for women. Crucially, however, no other significant main effects of sex or interactions between sex and stimulus type were observed for any of the remaining EPT ratings (see Fig 1B). Descriptive statistical data are provided in S2 Table.

In summary, women were more accurate than men in identifying the agent’s purpose in situations of accidental harm. All empathy ratings for intentional harms were higher in women than in men. Discomfort ratings for accidental harm were also higher in women. However, effect sizes were not even small in any of the main effects and interactions (the highest effect size was η^2^ = 0.003), reducing the statistical significance and the relevance of these sex differences. In addition, and more importantly, these results were not replicated in a smaller sample size, suggesting that significant *p*-values in our first analyses were inflated by the massive sample size.

### Moral judgment

#### Impersonal dilemma

**Fig 2:**
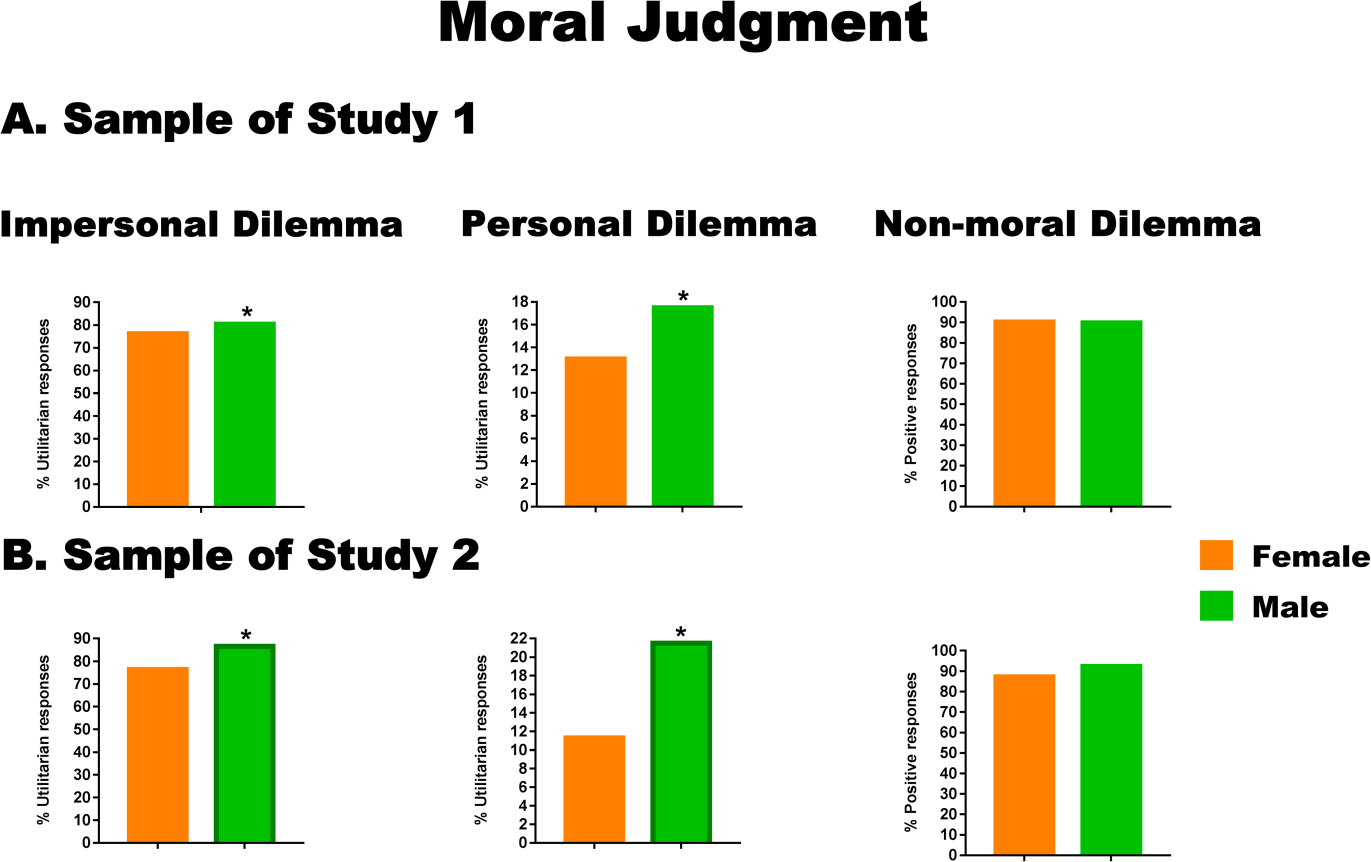
Significant sex differences in responses to moral and non-moral dilemmas. Asterisks indicate significant differences (*p* < .05). Differences with a small or higher effect size (Cramer’s V ≥ 0.1) are marked with a bold border.

Most participants (8,586, 79.5%) delivered a utilitarian response (i.e., yes, flip the switch), and 2,216 (20.5%) delivered a non-utilitarian response. Utilitarian responses to this dilemma were significantly more frequent in men than in women (X^2^ (1) = 29.19, *p* < .001, Cramer’s V = 0.05), although the effect size did not even reach the minimum criterion to be considered small (see Fig 2A).

#### Personal dilemma

A small proportion of participants (1,676, 15.5%) delivered a utilitarian response (i.e., yes, push the man). Most of them (9,126, 84.5%) delivered a non-utilitarian response (i.e., no, don’t push the man). Consistent with the previous analyses, utilitarian responses were significantly more frequent in men than in women (X^2^ (1) = 43.72, *p* < .001, Cramer’s V = 0.06). Nevertheless, the effect size did not even reach the minimum criterion to be considered small (see Fig 2A).

#### Non-moral dilemma

A total of 9,856 (91.2%) participants provided a positive response to the non-moral dilemma. No significant differences were found between men and women (X^2^ (1) = .16, *p* = .65, Cramer’s V = 0.004) (see Fig 2A).

Moral judgment data were also re-analyzed in the subsample from Study 2. Results were similar to those reported above (see Fig 2B), although significant sex differences in utilitarian responses to both moral dilemmas showed small effect sizes (Cramer’s V = 0.13 for both moral dilemmas). Details are provided in S2 Text.

### Re-analysis of empathy data with moral judgments as covariates

In light of the well-established relationship between empathy and morality (e.g. [49, 50–52]), we re-analyzed the empathy data using the responses to the personal dilemma as a covariate–for a similar approach see [16, 74]. Results showed that sex differences in comprehension of purpose for accidental harm (*F*(1, 10799) = 18.08, *p* < .001, η^2^ = 0.001) remained significant after covariance analysis, although a significant effect of moral judgment was observed (*F*(1, 10799) = 24.9, *p* < .001, η^2^ = 0.002). Group differences in empathic concern (*F*(1, 10799) = 26.22, *p* < .001, η^2^ = 0.002) and discomfort ratings (*F*(1, 10799) = 57.9, *p* < .001, η^2^ = 0.005) for intentional harms remained significant after adjusting for moral judgment. Significant group differences in discomfort ratings for accidental harm (*F*(1, 10799) = 18.27, *p* < .001, η^2^ = 0.001) were preserved, although a significant effect of moral judgment was also observed (*F*(1, 10799) = 19.02, *p* < .001, η^2^ = 0.001). Moreover, significant group differences in intention to hurt (*F*(1, 10799) = 32.60, *p* < .001, η^2^ = 0.003) and punishment ratings (F(1, 10799) = 32.22, *p* < .001, η^2^ = 0.002) for intentional pain situation were preserved after covariate analyses.

These covariance analyses were not performed in the subsample from Study 2 since, in that sample, no significant interactions between sex and stimulus type were observed for any of the EPT ratings.

### Are sex or moral judgment relevant predictors of empathy for pain?

We conducted multiple regression analyses to explore whether EPT performance was explained by sex or moral judgments. For these analyses, all measures yielding sex differences across stimulus type were framed as dependent variables. Given that all empathy measures for intentional harm differed among groups, we calculated a global empathy score based on the mean of such ratings (i.e., empathic concern, discomfort, intention to hurt, and punishment). We estimated three different multiple regression models featuring the following dependent variables: comprehension of agent’s intention in accidental harm, global empathy score, and discomfort ratings for accidental harm. The predictors in all regression models were sex and responses to the personal moral dilemma.

A first multiple regression model (*F*(2, 10799) = 22.94, *p <* .01, R^2^ = 0.004) showed that sex (beta = -0.04, *p* < .001, η^2^ = 0.001) and the response to the personal moral dilemma (beta = -0.04, *p* < .001, η^2^ = 0.002) were associated with comprehension of the agent’s intention is accidental harm scenarios. The second model (*F*(2, 10799) = 22.38, *p <* .01, R^2^ = 0.004) showed that sex (beta = -0.06, *p* < .001, η^2^ = 0.004) was associated with the global empathy score. Moral judgment was not a significant predictor (beta = 0.01, *p* = .49, η^2^ = 0.00004). The third model (*F*(2, 10799) = 17.53, *p <* .001, R^2^ = 0.003) evidenced that sex (beta = -0.04, *p* < .001, η^2^ = 0.001) and moral judgment (beta = 0.04, *p* < 0.01, η^2^ = 0.001) were associated with discomfort ratings for accidental harm.

We also performed multiple regression models in the subsample from Study 2. A first multiple regression model (*F*(2, 331) = 8.14, *p <* .01, R^2^ = 0.04), explaining 4% of the variance, showed that the response to the personal moral dilemma (beta = -0.18, *p* < .01, η^2^ = 0.03) was associated with comprehension of the agent’s intention in accidental harm. Sex was not significantly associated (beta = -0.08, *p* = .11, η^2^ = 0.007). The second model (*F*(2, 331) = 0.77, *p* = .46, R^2^ = 0.004) showed that neither sex (beta = -0.005, *p* = .91, η^2^ = 0.00003) nor moral judgment (beta = -0.06, *p* = .22 η^2^ = 0.004) was associated with the global empathy score for intentional harm. Similarly, the third model (*F*(2, 331) = 0.21, *p* = .8, R^2^ = 0.001) evidenced that sex (beta = -0.03, *p =* .54, η^2^ = 0.001) and moral judgment (beta = 0.01, *p* = .73, η^2^ = 0.0003) were not associated with discomfort ratings for accidental harm.

In addition, we performed three multiple regression analyses to explore whether sex or the differential effect of moral judgment for personal dilemmas (the score of the personal dilemma minus the score of the impersonal dilemma) was associated with the EPT performance. We considered the same three dependent variables (comprehension of agent’s intention in accidental harm, global empathy score, and discomfort ratings for accidental harm). Results showed that sex was significantly associated with all of these variables, while the differential effect of personal dilemmas was not significantly related with any of them (see details in S3 Text).

In brief, in the sample of Study 1, sex was associated with comprehension of the agent’s intention in accidental harm, the global empathy score for intentional harm, and the discomfort ratings for accidental harm. The response to the personal moral dilemma was related with intention comprehension and discomfort ratings for accidental harm. Although predictors associated with empathy in the three models showed significant *p*-values, effect sizes cannot even be considered small. Multiple regression analyses performed in the sample of Study 2 showed that sex was not significantly associated with any of the EPT measures. The response to the personal moral dilemma was associated with global comprehension of the agent’s intention in accidental harm scenarios, but with a small effect size. Overall, regression models do not fit the data well and predictors do not account for the variance in the dependent variables. Neither sex nor moral judgments were good predictors of EPT based on the effect sizes and standardized coefficients.

## Discussion of Study 1

The aim of this study was to address two main issues regarding alleged sex differences in empathy and morality: (a) the use of self-report measures which may be strongly biased by gender-role stereotypes, and (b) the scarcity of large population-based studies reporting effect sizes for the observed differences. To this end, we evaluated a large representative sample with a well-validated experimental task [15–19] eliciting automatic empathic responses to others’ pain, alongside two widely used moral dilemmas [67, 68]. Based on *p*-values, women were more accurate than men in ascertaining the agent’s intention during accidental harm and they also showed higher ratings in some empathy measures. Critically, however, none of the effect sizes reached values that could be even considered small, reducing the relevance of these sex differences. Moreover, we found that neither sex nor moral judgments were not good predictors of EPT results.

Participants in Study 1 completed an EPT, a very robust paradigm to induce empathic responses [50, 52, 56]. Importantly, the automaticity of the reactions thus elicited [59–61] renders EPT performance impervious to gender-relevant social norms and expectations. Our results showed that women had higher accuracy than men in comprehending the intention of an agent inflicting accidental harm. Moreover, all empathy ratings for intentional harm were higher in women than in men. Discomfort ratings for accidental harm were also higher in women. However, none of the effect sizes of main effects or interactions reached the criterion even for small (the highest effect size was η^2^ = 0.003), reducing the relevance of these sex differences. In addition, we failed to replicate these results in a smaller sample size (*n* = 334), which suggests that significant *p*-values are inflated by our large sample size. These results are consistent with those of previous studies using experimental designs [4, 12] that have reported no sex differences in empathy. Moreover, supporting our results, a previous study [76] reported no sex differences in empathy ratings when participants viewed video clips of individuals in pain. Finally, our results align with previous EPT research reporting no sex differences in hemodynamic responses [6, 14], eye gaze fixations, or pupil dilation [6]. In sum, it seems that empathy processing does not differ between sexes when assessed through instruments inducing more automatic responses.

Conversely, our results are inconsistent with those of several studies employing self-report measures [2–5, 20–22], which have reported greater empathy in women. It has been suggested [6, 7, 21, 23] that self-report empathy measures are strongly influenced by gender-relevant social norms and expectations (see below). As sensitivity and emotionality are part of the stereotypic feminine role, it is likely that women are more willing than men to present themselves as being empathetic, even if actual empathic responsiveness is similar between sexes [7]. Supporting our results, previous works [7, 10, 46] have posited that enhancements of empathy in females would be evident only when individuals are aware that they are being evaluated on an empathy-relevant dimension. These kinds of measures may activate the gender-role stereotype that women are more empathetic than men, thus increasing the levels of self-reported empathy in the former group. Such differences are not detected with experimental measures of empathy.

Incidentally, note that these results reflect one well-known problem of the psychological literature: the low rates of replicability across studies [32, 34, 37, 77, 78]. A recent study [32] showed that a large portion of replications did not reproduce originally reported results. This high rate of non-replication of results is partially a consequence of the strategy of claiming conclusive research findings solely on the basis of single studies based on formal statistical significance (typically a *p-value* of less than .05) [34]. Calculating and reporting effect sizes and confidence intervals are highly desirable practices to relieve the lack of replicability of psychological studies [24, 26]. Using larger samples is another recommendation for increasing the reproducibility and the precision of estimated effects [29, 35–37]. Larger samples more accurately represent the characteristics of the populations from which they are derived. For instance, the size of the confidence interval depends on the sample size and the standard deviation of the study groups [25]. If the sample size is large, this leads to “more confidence” and a narrower confidence interval. In this sense, our large-scale population-based study stands out by the robustness of its results. Larger samples, as the one analyzed here, increase the statistical power [34] and precision of the estimates concerning social cognitive processes.

Sex differences have also been reported in different aspects of morality [9, 47, 48, 79]. In particular, men have been observed to differentially favor utilitarian responses to moral dilemmas [47, 48]. Our results corroborate this pattern for both impersonal and personal moral dilemmas. These findings were replicated in the smaller sample of Study 2. However, these results must be interpreted with caution due to the small effect sizes. Moreover, it is worth considering that utilitarian responses to dilemmas as the ones employed here have not shown an association to genuine utilitarian judgments or attitudes in other contexts [80]. Future population-based studies including larger sets of more ecological moral dilemmas should explore sex differences to establish the meaningfulness of the present findings. In addition, utilitarian responses to moral dilemmas have been associated with traits and attitudes (e.g., psychopathy and rational egoism) [80, 81] that we did not assess in this study. Further research on sex differences in moral judgment should consider these factors.

All significant differences in empathy ratings survived covariation with responses to the personal dilemma. Moreover, a significant effect of moral judgment was observed on the comprehension of the agent’s intention during accidental harm, and the discomfort ratings for accidental harm. However, none of the effect sizes for these analyses could even be considered small. Although these results are consistent with previous studies reporting a direct relationship between empathy and morality (e.g. [49, 50–52]), the very small effect sizes dramatically reduces their meaningfulness. Moreover, our results suggest that the relationship between empathy and moral judgment are associated not only with crucial affective aspects of empathy (e.g., empathic concern), but also with secondary aspects such as the comprehension of intentionality behind accidental harm, and the discomfort ratings for accidental harm. Future studies should further explore the relationship between moral judgment and different aspects of empathy.

In addition, we conducted multiple regression analyses to explore whether EPT performance was explained by sex or moral judgment. In line with the results above, we found that sex and the response to the personal moral dilemma were associated with the comprehension of the agent’s intention in accidental harm and the discomfort ratings for accidental harm. Moreover, sex was associated with the empathy global score. These results were not replicated in Study 2. In addition, we explored whether the differential effect of moral judgment for personal dilemmas was associated with EPT performance. The results showed that this effect was not associated with any of the dependent variables. Overall, regression models do not fit the data well, and effect sizes and standardized coefficients showed that predictors do not account for the variance in the dependent variables. Thus, neither sex nor moral judgments seem to be good predictors of empathy, as assessed through the EPT.

In conclusion, although some significant sex differences emerged for the EPT in Study 1, effect sizes were minuscule, which undermines the statistical relevance of such differences. Hence, together with previous experimental studies [4, 12], our data indicates that sex does not play a crucial role in empathy. Moreover, neither sex nor moral judgments appear to be robust predictors of empathy ratings.

## Results of Study 2

This study aims at testing whether results obtained in Study 1 were replicated when self-report data are considered. To this end, a subsample of 334 individuals participating also completed a self-report questionnaire of empathy. No significant differences were observed between women and men in terms of age (*F*(1, 332) = 0.60, *p =* .43, η^2^ = 0.001).

### Self-reported empathy

**Fig 3:**
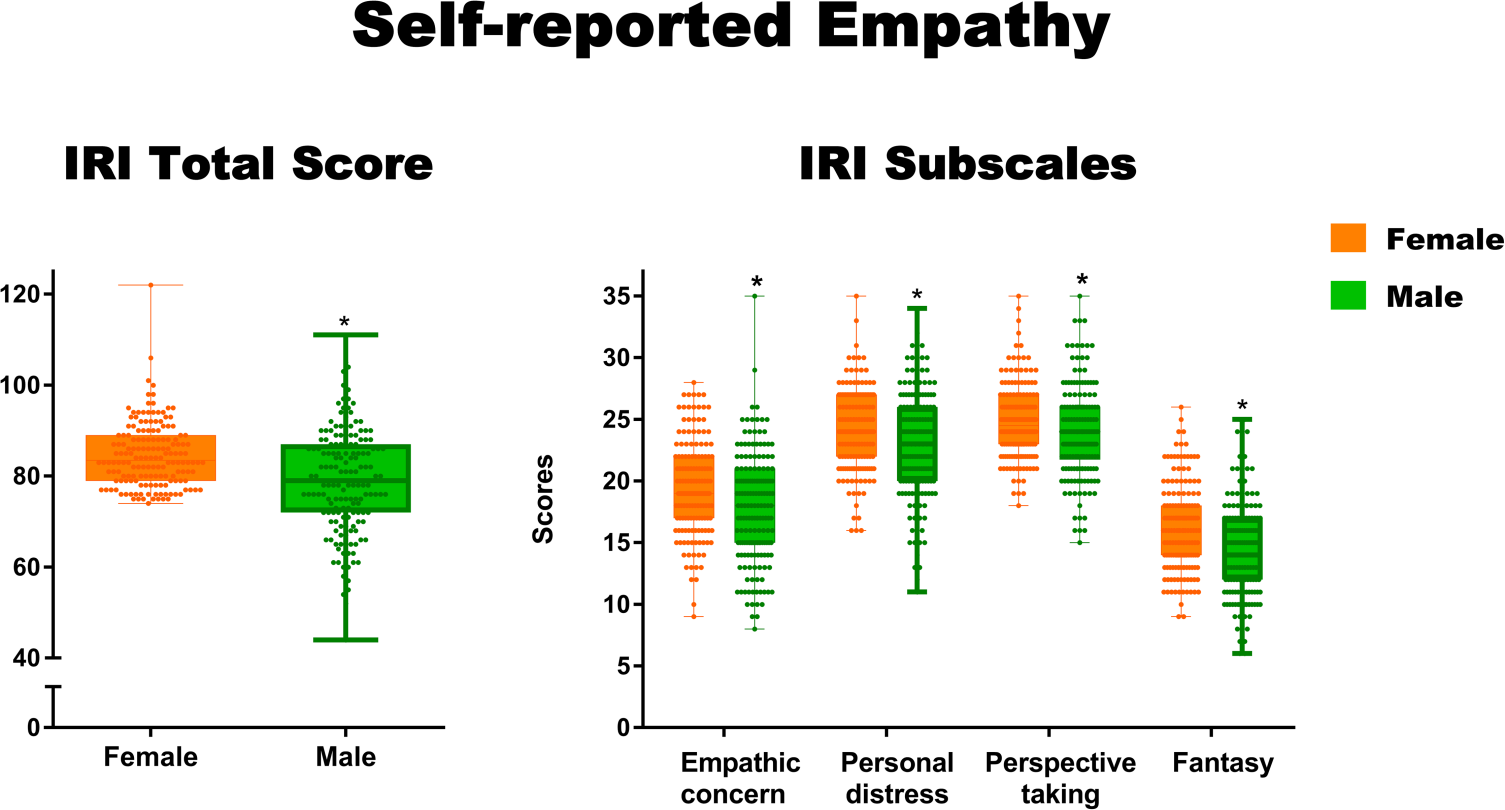
Significant sex differences in self-reported empathy. Asterisks indicate significant differences (*p* < .05). Differences with a small or higher effect size (η^2^ ≥ 0.01) are marked with a bold border.

Women showed significantly higher IRI total scores than men (*F*(1, 332) = 30.27, *p <* .001, η^2^ = 0.08). Moreover, women showed significantly higher scores than men in the subscales tapping empathic concern (*F*(1, 332) = 5.15, *p <* .01, η^2^ = 0.002), personal distress (*F*(1, 332) = 22.15, *p <* .001, η^2^ = 0.06), fantasy (*F*(1, 332) = 14.70, *p <* .001, η^2^ = 0.04), and perspective taking (*F*(1, 332) = 8.56, *p <* .01, η^2^ = 0.003)–see Fig 3. Although the *p*-values of all comparisons were statistically significant, effect sizes were relevant only for the total IRI score, and the personal distress and fantasy subscales.

### Re-analysis of self-reported empathy data with moral judgment as covariate

As in Study 1, we conducted multiple regression analyses to explore whether IRI scores were explained by sex or moral judgment. For these analyses, the items yielding significant sex differences were framed as dependent variables. Results showed significant group differences in the total IRI score (*F*(1, 331) = 29.90, *p* < .001, η^2^ = 0.08) were preserved after controlling for moral judgment. Moreover, significant sex differences in the subscales tapping empathic concern (*F*(1, 331) = 7.17, *p <* .01, η^2^ = 0.02), personal distress (*F*(1, 331) = 21.54, *p <* .001, η^2^ = 0.06), fantasy (*F*(1, 332) = 12.83, *p <* .001, η^2^ = 0.03), and perspective taking (*F*(1, 332) = 8.18, *p <* .01, η^2^ = 0.02) remained significant after covariance analyses. However, a significant effect of moral judgment (*p <* .001, η^2^ = 0.02) on empathic concern was also observed.

### Are sex or moral judgment relevant predictors of self-reported empathy?

We estimated five different models in which the total score and each of the IRI subscales were separately considered as dependent variables. The predictors in all regression models were sex and response to the personal moral dilemma.

A first multiple regression model (*F*(2, 331) = 15.11, *p <* .001, R^2^ = 0.08) showed that sex (beta = -0.29, *p* < .001, η^2^ = 0.08) was associated with the total IRI score. The personal moral dilemma (beta = 0.009, p = .85, η2 = 0.0001) was not associated with this measure. The second model (*F*(2, 331) = 7.01, *p* < .001, R^2^ = 0.04) showed that sex (beta = -0.14, *p* < .001, η^2^ = 0.02) and moral judgment (beta = -0.16, *p* < .001, η^2^ = 0.02) were significantly associated with empathic concern scores. The third model (*F*(2, 331) = 11.04, *p* < .001, R^2^ = 0.06) evidenced that sex (beta = -0.24, *p* < 0.001, η^2^ = 0.06) was a significant predictor of personal distress scores. Moral judgment (beta = -0.005, *p* = .92, η^2^ = 0.00003) was not a significant predictor.

The fourth model (*F*(2, 331) = 8.76, *p* > .001, R^2^ = 0.05) showed that sex (beta = -0.19, *p* = > .001, η^2^ = 0.03) was significantly associated with the scores in the fantasy subscale. Moral judgment (beta = -0.08, *p* = .09 η^2^ = 0.008) was not significantly associated. The last model (*F*(2, 331) = 4.29, *p* < .05, R^2^ = 0.01) evidenced that sex (beta = -0.15, *p* < .001, η^2^ = 0.02) was a significant predictor of perspective taking scores. Moral judgment (beta = -0.01, *p* = .8, η^2^ = 0.0001) was not related to scores in this subscale.

Summarizing, sex was a significant predictor of the all the IRI subscales and the instrument’s total score, with small and medium effect sizes, respectively. More precisely, effect sizes were small for empathic concern, fantasy, and perspective taking subscales, and medium for the personal distress subscale and the total IRI score. Moral judgment was not associated with any of the IRI scores.

## Discussion of Study 2

In this study, a subsample from Study 1 also completed a self-report questionnaire tapping various dimensions of empathy. Total IRI scores were higher for women than men, and the same was true of all the empathy subscales. Effect sizes were relevant only for the total IRI score and the personal distress and fantasy subscales. In addition, sex was a significant predictor of the total score and all the IRI subscales, with medium and small effect sizes, respectively. Irrelevant sex differences observed in Study 1 were not replicated by this subjective measure. This finding suggests that self-report empathy measures may be strongly biased by gender-relevant social stereotypes and expectations (see General discussion).

Women exhibited higher total IRI scores than men. In turn, the latter scored higher in the subscales tapping empathic concern, personal distress, fantasy, and perspective taking. However, effect sizes were relevant (small or higher) only for the total IRI score and the personal distress and fantasy subscales. These results align with previous reports of higher self-reported empathy in women (e.g., [2, 3, 4, 22, 27]). Moreover, significant differences and effect sizes reported here are consistent with those observed in previous applications of the IRI [20, 82, 83]. As was the case in other reports [82, 83], highest effect sizes were found for the total IRI score and the fantasy and personal distress subscales. Thus, women seem to present higher levels of both cognitive and affective components of self-reported empathy. In sum, we replicated the previously reported sex differences favoring women in self-reported empathy. However, although such differences have been systematically detected by measuring empathy with self-report questionnaires, it is worth noting that effect sizes reported here and in previous studies [20, 82, 83] range from small to moderate, suggesting a limited practical relevance.

All significant differences between women and men in the IRI scores were preserved after co-varying for moral judgment. Compatibly, multiple regression models revealed that sex was a significant predictor of the total IRI score and the personal distress subscale with medium effect sizes. Sex was also significantly associated with results from the empathic concern, fantasy, and perspective taking subscales, with small effect sizes. In addition, moral judgment had a significant effect on empathic concern. This finding is consistent with the results of previous studies [54, 55] showing that utilitarian moral judgment is associated with reduced empathic concern as measured by the IRI. Further studies should explore the relationship between utilitarian moral judgment and empathic concern in more population-representative samples, including experimental measures of empathic concern and a larger set of moral dilemmas.

Unlike Study 1, the results from this self-report study showed women as more empathic than men, with sex emerging as a significant predictor of scores on the IRI subscales. The discrepancies between both studies suggest that the detection of sex differences in empathy is strongly driven by the data collection method and associated processes involved. Whereas self-report instruments consistently yield sex differences, experimental paradigms of empathy fail to identify such differences.

## General discussion

Our two studies aimed to elucidate inconsistencies in the literature addressing sex differences in empathy. In line with available evidence, Study 1 showed no meaningful differences between sexes on an experimental EPT paradigm. Conversely, in Study 2, women scored higher than men in a self-report questionnaire of empathy. These results indicate that sex differences in empathy are not ubiquitous; rather, they emerge mainly under specific conditions. As previously proposed [6, 7, 21, 23], differential performance in experimental empathy tasks and self-report measures may reflect gender differences in how empathetic women and men would like to appear. Even if there were no intrinsic sex differences in empathy levels, women might tend to assume that they are expected to portray themselves as highly empathetic, thus favoring a widespread gender stereotype. Instead, men could refrain from describing themselves as emotional and sensitive, since this is not part of typical male stereotypes [44, 45]. Thus, questions in self-report instruments may prompt responses influenced by the participants’ identification with gender stereotypes [6, 7, 21, 23].

Unlike self-report questionnaires, empathic responses to others’ pain were not significantly and reliably different between women and men. EPT relies on more automatic responses [59–61]. These kinds of paradigms have been successful in the assessment of both healthy (e.g., [50, 52, 56, 61, 84]) and clinical (e.g., [15, 16–19, 62, 85]) populations, due to the robustness of pain in inducing empathic responses and its capacity to engage the neural circuit of empathy [58]. Hence, EPT paradigms seem more adequate than self-report questionnaires to assess a social complex phenomenon such as empathy.

Evidence from neuroimaging studies has shown that, at the neuroanatomical level, empathy processes engage a broad network including the insula, the anterior cingulate cortex, the supplementary motor area, the amygdala, the orbitofrontal cortex, and the temporoparietal junction [17, 50, 59–61, 86–88]. More specifically, perceiving an individual who intentionally hurts another person triggers an early boost in the amygdala [19], which plays a critical role in evaluating actual or potential threats [89–91]. In addition, witnessing a violent conflict of two individuals engaged in an aggressive interaction involves the activation of a set of regions associated with body and emotion perception, as well as social interaction [92], which includes the premotor cortex, the extra-striate body area, the fusiform gyrus, the insula, and the amygdala. Previous neuroimaging studies [6, 13, 14] suggest that activation of the neural network associated with empathy does not differ between women and men. However, future studies with larger samples should further explore whether neural correlates of empathy differ between sexes.

The relationship between empathy and morality has been well documented (e.g. [49, 50–52]). In a similar vein, Study 1 showed a significant effect of moral judgment on the comprehension of an agent’s intentions and the discomfort ratings for accidental harm. Similarly, Study 2 revealed a significant effect of moral judgment on empathic concern, but effect sizes were very small in both cases. Further studies are needed to understand the relationship between moral judgment and different measures of cognitive and affective empathy.

This is the first large-scale study assessing sex differences and gender roles in empathy and moral cognition. Given that our sample size is representative of individuals in our a priori defined population, and that our results are hence generalizable to the population addressed in the study, the latter may be considered as population-based research [93]. Despite their contributions, our studies feature some limitations. First, we assessed a population-based sample with the EPT task, but only a subsample of these participants completed the self-report questionnaire. Further studies should assess larger representative samples with self-report instruments to determine the actual impact of sex differences and their significance. Second, most responses came from Latin American countries. Although previous studies [15–19, 54, 62, 94, 95] have assessed Latin American populations with similar versions of the instruments employed here, future studies with representative samples from all over the world are needed to determine the generalizability of the present results. Third, though our findings suggest that sex differences in self-reported empathy may be explained by the participants’ identification with gender-role stereotypes, here we did not provide empirical support for this explanation. Further experimental studies on empathy and its relationship with gender-role stereotypes are needed to test this hypothesis. In addition, as we did not measure reaction times, future studies should explore sex differences in reaction times for empathy and moral cognition tasks and the relationship between reaction times and empathic and moral responses. Furthermore, our results showed that utilitarian responses to both impersonal and personal moral dilemmas were significantly more frequent in men than in women. However, given the small effect sizes, future population-based studies including larger sets of moral dilemmas should explore sex differences to establish the meaningfulness of these findings. Besides, further population-based studies should examine sex differences in empathy and moral judgment combining self-report, experimental, and neurophysiological measures to better characterize patterns of empathic responses in each sex. Last, though challenging, future studies on sex differences should also consider the inclusion of direct observation of empathic behaviors in order to test whether experimental measures predict actual social behavior [96].

## Conclusion

In conclusion, our results suggest that sex differences in empathy are highly dependent on the method of assessment. Higher levels of empathy in women were detected in self-reported empathy, while insignificant sex differences were observed in empathic responses to others’ pain. Moreover, we found that utilitarian responses to moral dilemmas were more frequent in men than in women. However, these results must be interpreted with caution due to the small effect sizes. These findings highlight the need for further studies on sex differences in empathy and moral judgment, including population-based samples, multiple measures, and documentation of effect sizes. Besides, future studies should control for social and cultural variables potentially related to empathic responses and gender-role stereotypes. These considerations may extend our knowledge of these complex social phenomena.

## Supporting information

S1 Text Results from the empathy task analyzed with non-parametric tests (Study 1). (DOC)

S2 Text Results from the moral judgment task in the Study 2 subsample. (DOC)

S3 Text Results of multiple regression analyses including a differential effect of personal dilemmas as predictor (Study 1). (DOC)

S1 Fig Distribution of participants’ nationalities for sample 1 and sample 2. (TIF)

S1 Table Descriptive data from the empathy-for-pain task (Study 1 sample). (DOC)

S2 Table Descriptive data from the empathy-for-pain task (Study 2 sample). (DOC)
