# Supplementary material for: Men, women…who cares? A population-based study on sex differences and gender roles in empathy and moral cognition
Source: PLoS One. 2017 Jun 20;12(6):e0179336. doi: 10.1371/journal.pone.0179336 (PMC5478130; doi:10.1371/journal.pone.0179336)
Supplement: S1 Text — (DOC) [file pone.0179336.s001.doc]

**Men, women…who cares? A population-based study on sex differences and gender roles in empathy and moral cognition**



Sandra Baez, Daniel Flichtentrei, María Prats, Ricardo Mastandueno, Adolfo M. García, Marcelo Cetkovich, Agustín Ibáñez

**S1 Text. N**on-parametric Kruskal-Wallis tests revealed that women were more accurate than men in ascertaining the agent’s intention situations of accidental harm (*H* (1, 10802) = 12.22, *p < .*001, η^2^ = 0.000001). Moreover, women provided higher empathic concern ratings than men for intentional harm (*H* (1, 10802) = 21.09, *p < .*001, η^2^ = 0.001).Women also showed higher discomfort ratings than men for intentional (*H* (1, 10802) = 57.369, *p < .*001, η^2^ = 0.004) and accidental (*H* (1, 10802) = 30.05, *p < .*001, η^2^ = 0.001) harm. With respect to intention to harm, ratings for intentional harms were higher in women relative to men (*H* (1, 10802) = 29.77, *p < .*001, η^2^ = 0.002). Finally, women gave higher punishment ratings than men for intentional harm (*H* (1, 10802) = 31.56, *p < .*001, η^2^ = 0.002).
