# Supplementary material for: Men, women…who cares? A population-based study on sex differences and gender roles in empathy and moral cognition
Source: PLoS One. 2017 Jun 20;12(6):e0179336. doi: 10.1371/journal.pone.0179336 (PMC5478130; doi:10.1371/journal.pone.0179336)
Supplement: S2 Text — (DOC) [file pone.0179336.s002.doc]

**Men, women…who cares? A population-based study on sex differences and gender roles in empathy and moral cognition**



Sandra Baez, Daniel Flichtentrei, María Prats, Ricardo Mastandueno, Adolfo M. García, Marcelo Cetkovich, Agustín Ibáñez

**S2 Text. Moral judgment in the Study 2 subsample**

**Impersonal dilemma.** 276 (83.6%) participants delivered a utilitarian response while 58 (17.4%) participants delivered a non-utilitarian response. Utilitarian responses to this dilemma were significantly more frequent in men than in women (X^2^ (1) = 6.06, *p* < 0.05, Cramer’s V = 0.13), with a small effect size.

**Personal dilemma.** 56 (16.9%) participants delivered a utilitarian response and 278 (84.1%) delivered a non-utilitarian response. Consistent with the prior analyses, utilitarian responses were significantly more frequent in men than in women (X^2^ (1) = 6.19, *p* < 0.05, Cramer’s V = 0.13), with small effect size.

**Non-moral dilemma.** 304 (91%) participants provided a positive response to the non-moral dilemma. No significant differences were found between men and women (X^2^ (1) = 2.67, *p* = 0.10, Cramer’s V = 0.08).
