# Supplementary material for: Men, women…who cares? A population-based study on sex differences and gender roles in empathy and moral cognition
Source: PLoS One. 2017 Jun 20;12(6):e0179336. doi: 10.1371/journal.pone.0179336 (PMC5478130; doi:10.1371/journal.pone.0179336)
Supplement: S3 Text — (DOC) [file pone.0179336.s003.doc]

**Men, women…who cares? A population-based study on sex differences and gender roles in empathy and moral cognition**



Sandra Baez, Daniel Flichtentrei, María Prats, Ricardo Mastandueno, Adolfo M. García, Marcelo Cetkovich, Agustín Ibáñez

**S3 Text.** A first multiple regression model (*F*(2, 10799) = 9.89, *p <* .01, R^2^ = 0.02) showed that sex (beta = -0.04, *p* < .001, η^2^ = 0.01) was associated with comprehension of the agent’s intention is accidental harm scenarios. The differential effect of moral judgment of the personal dilemma (beta = 0.08, *p* = 0.72, η^2^ = 0.00002) was not a significant predictor. The second model (*F*(2, 10799) = 20.60, *p <* .01, R^2^ = 0.004) showed that sex (beta = -0.06, *p* < .001, η^2^ = 0.004) was associated with the global empathy score. The differential effect of moral judgment of the personal dilemma was not a significant predictor (beta = 0.01, *p* = 0.92, η^2^ = 0.00004). The third model (*F*(2, 10799) = 8.73, *p <* .001, R^2^ = 0.002) evidenced that sex (beta = -0.03, *p* < .001, η^2^ = 0.0001) was associated with discomfort ratings for accidental harm. The differential effect of moral judgment of the personal dilemma was not associated with the dependent variable (beta = 0.009, *p* = 0.33, η^2^ = 0.0003).
