# Supplementary figures and images for: Men, women…who cares? A population-based study on sex differences and gender roles in empathy and moral cognition
Source: PLoS One. 2017 Jun 20;12(6):e0179336. doi: 10.1371/journal.pone.0179336 (PMC5478130; doi:10.1371/journal.pone.0179336)

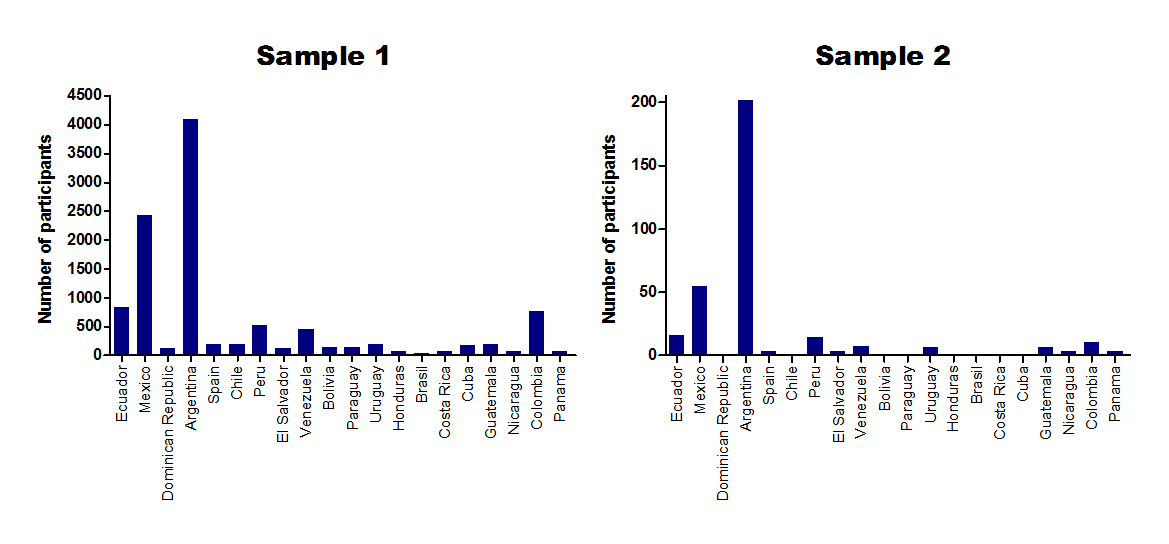

Supplement: S1 Fig — (TIF) [file pone.0179336.s004.tif]
