# Supplementary material for: Men, women…who cares? A population-based study on sex differences and gender roles in empathy and moral cognition
Source: PLoS One. 2017 Jun 20;12(6):e0179336. doi: 10.1371/journal.pone.0179336 (PMC5478130; doi:10.1371/journal.pone.0179336)
Supplement: S1 Table — (DOC) [file pone.0179336.s005.doc]

**S1 Table.** Descriptive data from the empathy-for-pain task (Study 1 sample)

|  |  | **Female**  **mean ± SD [95%CI]** | **Male**  **mean ± SD [95%CI]** |
| --- | --- | --- | --- |
|  | Purpose comprehension | 96.22 ± 12.14 [95.90-96.55] | 95.24 ± 14.21[94.86-95.62] |
| **Intentional harm** | Empathic concern | 72.32 ± 24.47 [71.67-72.98] | 69.85 ± 25.90 [69.16-70.53] |
|  | Discomfort | 77.76 ± 21.66 [77.18-78.34] | 74.44 ± 23.43 [73.82-75.06] |
|  | Intention to hurt | 80.66 ± 19.78 [80.13-81.19] | 78.41 ± 21.18 [77.84-78.97] |
|  | Punishment | 78.98 ± 21.03 [78.41-79.54] | 76.65 ± 22.28 [76.06-77.25] |
|  | Purpose comprehension | 96.26 ± 10.73 [95.98-96.55] | 95.22 ± 12.79 [94.88-95.56] |
| **Accidental harm** | Empathic concern | 27.67 ± 23.45 [27.04-28.29] | 26.60 ± 23.95 [25.97-27.24] |
|  | Discomfort | 22.13 ± 20.87 [21.57-22.69] | 20.52± 20.83 [19.97-21.08] |
|  | Intention to hurt | 9.23 ± 12.78 [8.89-9.57] | 9.76 ± 14.07 [9.38-10.13] |
|  | Punishment | 8.53 ± 12.40 [8.20-8.86] | 13.46 ± 13.46 [8.56-9.28] |
|  | Purpose comprehension | 71.84 ± 24.74 [71.23-72.45] | 72.60 ± 23.41 [71.98-73.22] |
| **Neutral situations** | Empathic concern | 9.32 ± 13.50 [8.96-9.68] | 9.64 ± 14.16 [9.26-10.02] |
|  | Discomfort | 8.91 ± 13.18 [8.56-9.27] | 9.03 ± 13.55 [8.67-9.39] |
|  | Intention to hurt | 7.37 ± 11.89 [7.05-7.68] | 7.96 ± 13.02 [7.61-8.31] |
|  | Punishment | 6.25 ± 10.89 [5.95-6.54] | 9.28 ± 6.89 [6.57-7.22] |
