# Supplementary material for: Men, women…who cares? A population-based study on sex differences and gender roles in empathy and moral cognition
Source: PLoS One. 2017 Jun 20;12(6):e0179336. doi: 10.1371/journal.pone.0179336 (PMC5478130; doi:10.1371/journal.pone.0179336)
Supplement: S2 Table — (DOC) [file pone.0179336.s006.doc]

**S2 Table.** Descriptive data from the empathy-for-pain task (Study 2 sample)

|  |  | **Female**  **mean ± SD [95%CI]** | **Male**  **mean ± SD [95%CI]** |
| --- | --- | --- | --- |
|  | Purpose comprehension | 97.40 ± 8.12 [96.15-98.66] | 94.85 ± 14.87 [92.60-97.10] |
| **Intentional harm** | Empathic concern | 73.58 ± 24.39 [69.82-77.34] | 74.11 ± 24.78 [70.36-77.86] |
|  | Discomfort | 79.66 ± 19.65 [76.63-82.69] | 78.71 ± 20.69 [75.58-81.85] |
|  | Intention to hurt | 80.71 ± 18.89 [77.79-83.62] | 80.71 ± 20.24 [77.64-83.78] |
|  | Punishment | 80.82 ± 18.58 [77.96-83.69] | 78.92 ± 22.14 [75.56-82.27] |
|  | Purpose comprehension | 96.79 ± 10.04 [95.25-98.34] | 93.82 ± 16.04 [91.39-95.25] |
| **Accidental harm** | Empathic concern | 30.27 ± 22.75 [26.77-33.77] | 28.89 ± 22.93 [25.42-32.37] |
|  | Discomfort | 27.15 ± 20.86 [23.94-30.37] | 25.78 ± 22.69 [22.35-29.22] |
|  | Intention to hurt | 10.81 ± 12.21 [8.93-12.69] | 12.62 ± 17.37 [9.98-15.25] |
|  | Punishment | 9.72 ± 12.00 [7.87-11.57] | 11.69 ± 16.99 [9.12-14.26] |
|  | Purpose comprehension | 67.91 ± 24.44 [65.94-73.48] | 67.25 ± 22.49 [63.83-70.66] |
| **Neutral situations** | Empathic concern | 11.60 ± 13.39 [9.53-13.66] | 11.12 ± 17.50 [8.47-13.77] |
|  | Discomfort | 11.59 ± 13.49 [9.51-13.67] | 11.94 ± 18.24 [9.1-14.71] |
|  | Intention to hurt | 9.13 ± 11.69 [7.32-10.93] | 10.11 ± 16.91 [7.55-12.68] |
|  | Punishment | 7.83 ± 11.17 [6.11-9.55] | 9.24 ± 16.74 [6.70-11.77] |
